# Measuring the Dash

**Published:** 2005-06-15

**Authors:** Lynne S. Wilcox

Individuals in the United States paradoxically long for and dread old age. The alternative to growing old is an outcome few of us would prefer. But aging seems inevitably associated with decline. Independent choices, a hallmark of adulthood, can be lost between one day and the next with a heart attack, a stroke, or a fractured hip. Senior citizen discounts notwithstanding, our culture does not offer a valued place for elders. It becomes easy to focus on what we will certainly lose rather than on any uncertain gains associated with aging.

And yet, we have examples of thriving elders. Former President Jimmy Carter won the Nobel Peace Prize in 2002 at age 78. Dr Benjamin Mays, a president of Morehouse College and mentor to Martin Luther King, Jr, and Andrew Young, published his autobiography *Born to Rebel* at 76. Celia Cruz, the Cuban American singer called the "Queen of Salsa," whose songs earned 22 gold records, continued performing and recording until her death at 78.

These people not only lived long, they lived well. How does one flourish over eight or nine decades? Joan Erikson was the wife and professional partner of the developmental psychologist Erik Erikson. Together they characterized eight stages of human development, from developing trust in infancy to encouraging generativity in old age (1). At age 90, soon after Erik's death, Joan began describing a ninth stage of wisdom that continued to include autonomy and growth. "But only if we've taken care of our bodies," she stressed. "The consistent care necessary to keep the body machinery functioning in spite of age and deterioration is mandatory" (2). This issue of *Preventing Chronic Disease* explores the role of public health in healthy aging.

Several authors describe current challenges in healthy aging in the United States. Gohdes and colleagues discuss age-related eye diseases; low vision and blindness rise dramatically with age in every race and ethnic group (3). Lai et al found that recovery from stroke in a Kansas population was related to sex, in part because prestroke functioning was lower in women compared with men in the study (4). Shenson and colleagues developed an index on the use of preventive services (Pap test, colon cancer screening, mammogram, and influenza and pneumococcal vaccines) in older Americans (5). The percentage of the population that achieved age- and sex-appropriate completion of these services ranged from 21% of women aged 50 to 64 years to 40% of men aged 65 years and older (5).

Stimpson et al used data from the Hispanic Established Population for the Epidemiologic Studies of the Elderly to examine concordance of chronic disease between older Mexican American spouses. Their findings of similarities in disease between spouses led them to recommend including spousal diagnoses in patient medical histories (6).

State public health programs have a role in addressing these and related concerns of aging. As always, health surveillance is a major tool, indicated in this issue by the report from the Association of State and Territorial Chronic Disease Program Directors describing a mapping study of stakeholders' attitudes toward state roles in healthy aging and less prevalent chronic diseases (7). Maylahn and coauthors examine the use of quality-of-life indicators in older citizens, and Moriarty et al provide information on the development of these indicators (8,9).

Concerns about an aging population are not limited to the United States or even to the most industrialized countries. China, India, and many other nations face increasingly higher proportions of elders in their populations. Hawkins describes the efforts of the Global Ageing Research Network (GARNet) to establish common measures of healthy aging for intracountry and intercountry comparisons (10). A world growing older may see changes in economics, policies, and social securities not experienced by recent generations. This is a critical test of public health's ability to integrate multiple domains to improve the health of citizens around the globe.

Joan Anderson's book *A Walk on the Beach* describes her friendship with Joan Erikson in the last years of Erikson's life (2). She provides an account of the two women wandering through an old cemetery on Cape Cod in Massachusetts. Erikson lifts her walking cane and places it on the dates, *1860–1928*, listed on a headstone. "It's all about the dash," she says (2,11). The recorded dates of birth and death are the least important aspects of a life. The dash speaks for all the time one spends on earth.
